# Innate and adaptive anti-viral immune responses in MS patients treated with interferon-beta

**DOI:** 10.1186/1742-4690-8-S1-A214

**Published:** 2011-06-06

**Authors:** Thor Petersen, Anné Møller-Larsen, Steffen Thiel, Troels K Hansen, Svend Ellermann-Eriksen, Tomasz Brudek, Tove Christensen

**Affiliations:** 1 Department of Neurology, Aarhus University Hospital, Aarhus, DK-8000 C, Denmark; 2 Department of Medical Microbiology and Immunology, Aarhus University, Aarhus, DK-8000 C, Denmark; 3 Department of Endocrinology, Aarhus University Hospital, Aarhus, DK-8000 C, Denmark; 4 Department of Clinical Microbiology, Aarhus University Hospital, Skejby, DK-8200 N, Denmark

## Background

Interferon-beta (IFN-β) has both immuno-modulating and anti-viral effects. In a longitudinal study of multiple sclerosis (MS) patients undergoing interferon-beta therapy, we have performed a comprehensive study of factors in the innate and adaptive immune response to the two types of virus associated with MS: human endogenous retroviruses (HERVs), and herpesviruses.

## Materials and methods

Anti-viral antibodies towards HERVs and herpesviruses were assayed using TRIFMA or ELISA. Cytokine profiling was performed using the Luminex-system. Factors in the lectin complement activation pathway were assayed using TRIFMA.

## Results

We demonstrate significant decreases in anti-Envelope antibody reactivity for the two closely related Gammaretroviral HERVs, HERV-H and HERV-W, as a consequence of IFN-β therapy, closely linked to efficacy of therapy/low disease activity. We also found strong indications of a protective effect of high levels of two components in the innate pathogen-associated molecular pattern recognition: mannan-binding lectin (MBL), and MASP-3.

Serum levels of typical Th1- and Th2- related, MS-relevant cytokines were also monitored. We found no overall changes in Th1/Th2 ratios.

## Conclusions

Our results support that IFN-β exerts effects on immune response to HERV-H/HERV-W, and that this antiviral response may play a role in MS development. Components in the immune response to HERVs have potential as biomarkers for disease activity.

